# A Reduce and Replace Strategy for Suppressing Vector-Borne Diseases: Insights from a Deterministic Model

**DOI:** 10.1371/journal.pone.0073233

**Published:** 2013-09-04

**Authors:** Michael A. Robert, Kenichi Okamoto, Alun L. Lloyd, Fred Gould

**Affiliations:** 1 Department of Mathematics and Biomathematics Graduate Program, North Carolina State University, Raleigh, North Carolina, United States of America; 2 Department of Entomology, North Carolina State University, Raleigh, North Carolina, United States of America; 3 Fogarty International Center, National Institutes of Health, Bethesda, Maryland, United States of America; National University of Singapore, Singapore

## Abstract

Genetic approaches for controlling disease vectors have aimed either to reduce wild-type populations or to replace wild-type populations with insects that cannot transmit pathogens. Here, we propose a Reduce and Replace (R&R) strategy in which released insects have both female-killing and anti-pathogen genes. We develop a mathematical model to numerically explore release strategies involving an R&R strain of the dengue vector *Aedes aegypti*. We show that repeated R&R releases may lead to a temporary decrease in mosquito population density and, in the absence of fitness costs associated with the anti-pathogen gene, a long-term decrease in competent vector population density. We find that R&R releases more rapidly reduce the transient and long-term competent vector densities than female-killing releases alone. We show that releases including R&R females lead to greater reduction in competent vector density than male-only releases. The magnitude of reduction in total and competent vectors depends upon the release ratio, release duration, and whether females are included in releases. Even when the anti-pathogen allele has a fitness cost, R&R releases lead to greater reduction in competent vectors than female-killing releases during the release period; however, continued releases are needed to maintain low density of competent vectors long-term. We discuss the results of the model as motivation for more detailed studies of R&R strategies.

## Introduction

In recent decades, a number of vector-borne diseases have experienced a global resurgence due to changes in disease management strategies, development of insecticide and drug resistance, changes in social behaviors, pathogen evolution, and other factors [1], [2]. For some vector-borne diseases there are no vaccines or prophylactic drugs. This leaves vector control as the primary method for disease suppression [3], [4]. Traditional forms of vector control, such as source reduction and insecticide treatments, have sometimes been successful at reducing vector densities, but it is difficult to maintain these control programs indefinitely, and despite widespread applications of such programs, vector-borne diseases remain endemic in many regions of the world [1], [5]–[7].

A number of alternative methods of vector control have been proposed, including genetic pest management (GPM) approaches that aim to either reduce or eliminate the vector population or replace the native population with insects that cannot transmit a pathogen [8], [9]. Genetic constructs have been proposed and explored theoretically for either reduction [10]–[14] or replacement [15]–[20] strategies, and the transgenes necessary for both types of strategies have been developed for some species [21]–[24]. GPM approaches are being considered for a number of disease vectors, but one species for which there has been tangible progress is *Aedes aegypti,* the principal vector of dengue fever [25]. For this reason, we focus on the *Ae. aegypti* system throughout this paper.

One population reduction strategy that has been built and tested in *Ae. aegypti* is based on transgenes that cause female-specific mortality [21], [26], [27]. In order to rear large numbers of the transgenic mosquitoes for releases, the transgene that codes for lethality is engineered to have conditional expression [12], [14]. In one current transgenic strain of *Ae. aegypti*, the lethal transgenes are repressed when mosquitoes are reared on a diet containing tetracycline [21]. In the absence of tetracycline, females that inherit the transgene are incapable of surviving to reproduce, while males inheriting the gene will survive, and their offspring can inherit the female-specific lethality gene. Repeated releases of this *Ae. aegypti* Female-Killing (FK) strain into large laboratory cages with wild-type *Ae. aegypti* populations resulted in extinctions [26]. In a similar experiment in outdoor cages in Mexico there was a reduction in mosquito density but extinction did not occur [27].

Several mathematical models have been developed to assess the feasibility of FK strategies. Simple models predict that repeated releases of FK mosquitoes into wild-type populations will cause extinction in a time frame of about 1–2 years under ideal conditions [28]. Because males pass on the FK gene to their offspring, this strategy is expected to be more effective in reducing a population than strategies involving mortality of all offspring (*e.g.*, the classical Sterile Insect Technique) [29], [30]. More detailed models demonstrate that biological complexities not addressed by the cage experiments, particularly density-dependent population regulation and spatial heterogeneity, can affect the success of FK and other population reduction strategies [13], [29]–[34].

A failure of FK strategies to entirely eliminate a native population of disease vectors could have severe economic and public health consequences. Maintenance of the wild-type population at low levels would require the continuous production and release of mosquitoes. If releases were stopped after a number of years, the vector population would recover, potentially causing a severe epidemic in a human population lacking herd immunity.

Until now, most proposed transgenic strategies have focused on either vector reduction or vector replacement. In this paper, we propose a Reduce and Replace (R&R) strategy in which released insects have both an FK gene and an anti-pathogen (AP) gene. We theoretically assess the efficacy of potential R&R release scenarios with a system of ordinary differential equations that models both the population dynamics and population genetics of an *Ae. aegypti* population. We model R&R releases in a population for which vector elimination is difficult due to the strength of density-dependent larval population regulation. Because elimination in such a population is unlikely, it would be an ideal candidate for an R&R strategy. We show that it would be possible to reduce a population in a realistic time frame while ensuring that, when releases end, the reestablished population would have a low frequency of competent vectors.

## Methods

The R&R strain we consider in this paper has one FK allele and one AP allele located on two different chromosomes. We track modified and wild-type alleles (‘K’ and ‘k’ for FK, ‘A’ and ‘a’ for AP, respectively) at two independently segregating loci, which results in nine possible genotypes (Table 1). We divide the population into juveniles, adult males, and adult females. The juvenile class includes larvae and pupae in one class; egg production is modeled implicitly. Each of the three classes is further divided by genotype. We let *J_i_*(*t*), *M_i_*(*t*), and *F_i_*(*t*) represent the density of juveniles, adult males, and adult females, respectively, of genotype *i* at time *t*. Our model tracks matings between adults, births of juveniles, adult emergence, and deaths.

**Table 1:** Properties of genotypes resulting from R&R releases.

*i*	Genotype	*w_i_*	*γ_i_*
1	KKAA	(1-*c_A_*)(1-*c_K_*)	0*
2	KkAA	(1-*c_A_*)(1-0.5*c_K_*)	0
3	kkAA	(1-*c_A_*)	1
4	KKAa	(1-0.5*c_A_*)(1-*c_K_*)	0
5	KkAa	(1-0.5*c_A_*)(1-0.5*c_K_*)	0
6	kkAa	(1-0.5*c_A_*)	1
7	KKaa	(1-*c_K_*)	0*
8	Kkaa	(1-0.5*c_K_*)	0
9	kkaa	1	1

Possible genotypes resulting from R&R releases with corresponding fitness values (*w*_i_) and female viability coefficients (*γ*_i_).

* These females are, however, viable when released as adults due to conditional lethality.

We assume random mating between adult males and females and that inheritance is Mendelian. The rate at which females produce viable larvae of genotype *i* is given by *B_i_*(*t*), where
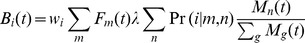



Here, *λ* is the per capita rate at which females produce larvae, Pr(*i*|*m,n*) is the Mendelian probability that an offspring of genotype *i* arises from a mating between a female of genotype *m* and a male of genotype *n*, and *w_i_* is the fitness of an offspring of genotype *i* relative to that of wild-type offspring (fitness is defined here as the fraction of eggs that survive and hatch into larvae). Throughout, we assume that fitness costs are additive at a given locus and multiplicative across loci. We define the fitness cost associated with an individual that is homozygous for the FK allele to be *c_K_* and for the AP allele to be *c_A_*. The resulting fitness values for each genotype are listed in Table 1. Although we assume additive fitness costs that reduce egg viability, the model can easily be adapted to consider other types of fitness costs (*e.g.,* dominant or recessive), as well as fitness disadvantages at other life stages (*e.g.,* mating or adult viability).

Juvenile mortality is assumed to have both density-independent and density-dependent components, represented as per capita mortality rates *µ_J_* and *αJ^β^* ^-1^, respectively. Here *J* is the total density of juveniles, and α and *β* are parameters that determine the strength of density-dependent mortality, and along with other parameters, the equilibrium population density [35], [36]. The strength of density dependence refers to how quickly the population returns to equilibrium after perturbation away from equilibrium density: higher strengths of density-dependent mortality (i.e., larger values of *β*), lead to a more rapid rate of recovery. While the model could be altered to consider density-dependent effects in other life stages, we choose to consider only larval density-dependent mortality because of the observed relationship between high-density *Ae. aegypti* larval populations and increased mortality [37]–[40]. Juveniles emerge as mature adults at a per capita rate *ν*. We assume a 1∶1 sex ratio at birth so that, in the absence of FK effects, one-half of the juveniles that emerge to adulthood are female and the other half male. Lethality induced by the FK allele is assumed to occur as adults emerge [13], [21]. We multiply the rate of emergence of female adults by a binary constant *γ_i_*, where, *γ_i_* = 1 for viable genotypes and γ_i_ = 0 otherwise (see Table 1). Adult males and viable females die at per capita rates *µ_M_* and *µ*_F_, respectively. Adult males and females of genotype *i* are introduced at rates 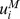
and 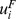, respectively. We consider the introductions of two of the listed genotypes: FK only (KKaa, *i* = 7) and R&R (KKAA, *i* = 1). Note that releases of transgenic adult females are possible because of conditional lethality: released females that are fed on a diet containing tetracycline as juveniles do not experience additional mortality due to carrying transgenes (in the absence of fitness costs) [12], [14], [21].

We simulate releases into a wild-type population that is at equilibrium. Continuous releases of males of genotype *i* occur at a rate 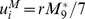, where 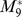 is the equilibrium wild-type male population density, *r* is ratio of transgenic adults released each week to the wild-type adult males in the population at the beginning of the release, and the factor 7 converts from a weekly to a daily rate. Continuous releases of females are defined similarly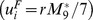. We note that the same number of transgenic individuals is released each week regardless of changes in the number of individuals in the mosquito population over time.

We obtain the system of ordinary differential equations, where *i* represents the genotype of each class
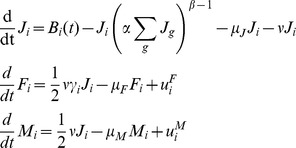



Since the only viable female genotypes are those with no FK alleles and released females, we need not track all female genotypes. Table 2 lists all model parameters described in this section with their default values.

**Table 2:** Model parameters.

Parameter	Description	Default Value	Reference
*µ_J_*	Density-independent juvenile mortality rate (per capita)	0.03 day^−1^	[41]
*µ_M_*	Male mortality rate (per capita)	0.28 day^−1^	[42], [43]
*µ_F_*	Female mortality rate (per capita)	0.10 day^−1^	[42], [43]
*λ*	Average rate of larval production by females (per capita)	8 day^−1^	[44], [45]
*ν*	Rate of emergence to adulthood (per capita)	0.14 day^−1^	[42]
*α,β*	Density dependence parameters	2×10^−4^, 3.4	–
*c*_A_	Fitness cost associated with anti-pathogen allele	0	–
*c*_K_	Fitness cost associated with female-killing allele	0	–
*w_i_*	Fitness of genotype *i*	See Table 1	–
*γ_i_*	Female viability coefficient of genotype *i*	See Table 1	–
*r*	Weekly release ratio of R&R individuals to wild-type males at the pre-release equilibrium	varied	–
*T*	Duration of release	varied (day)	–

Description of model parameters with default values and references for default values.

## Results

Due to the complexity of the system, this model does not lend itself to algebraic analysis. We can, however, obtain the wild-type population steady state solutions; we use these values as the initial values in our simulations. For the derivation of these values, we refer the reader to Text S1. We conducted numerical simulations using the ordinary differential equation solver ode15s in Matlab (Version 7.12, Mathworks, Natick, Massachusetts, U.S.A.) to explore the model. We begin by presenting the general dynamics of the R&R system and evaluating the long-term impact of R&R releases compared to FK releases. We then present a variety of release scenarios that result from varying release ratios and release durations, as well as from considering female-only and bi-sex releases. Finally, we assess the effects of fitness costs associated with the AP gene.

Because an R&R strategy seeks to decrease the number of disease cases, we are specifically interested in the effects that releases have on the overall density of adult females capable of vectoring the pathogen (hereafter referred to as competent vectors). We present the trajectories of the density of all adult females and competent vectors relative to the initial density of the adult female population. We also present time series for the frequencies of the FK and AP alleles in the juvenile population. Illustrating allele frequency dynamics in the juvenile population allows us to disentangle the contribution of released individuals in the adult population where allele frequencies are highly elevated during releases.

### Dynamics of the R&R System

**Figure 1:**
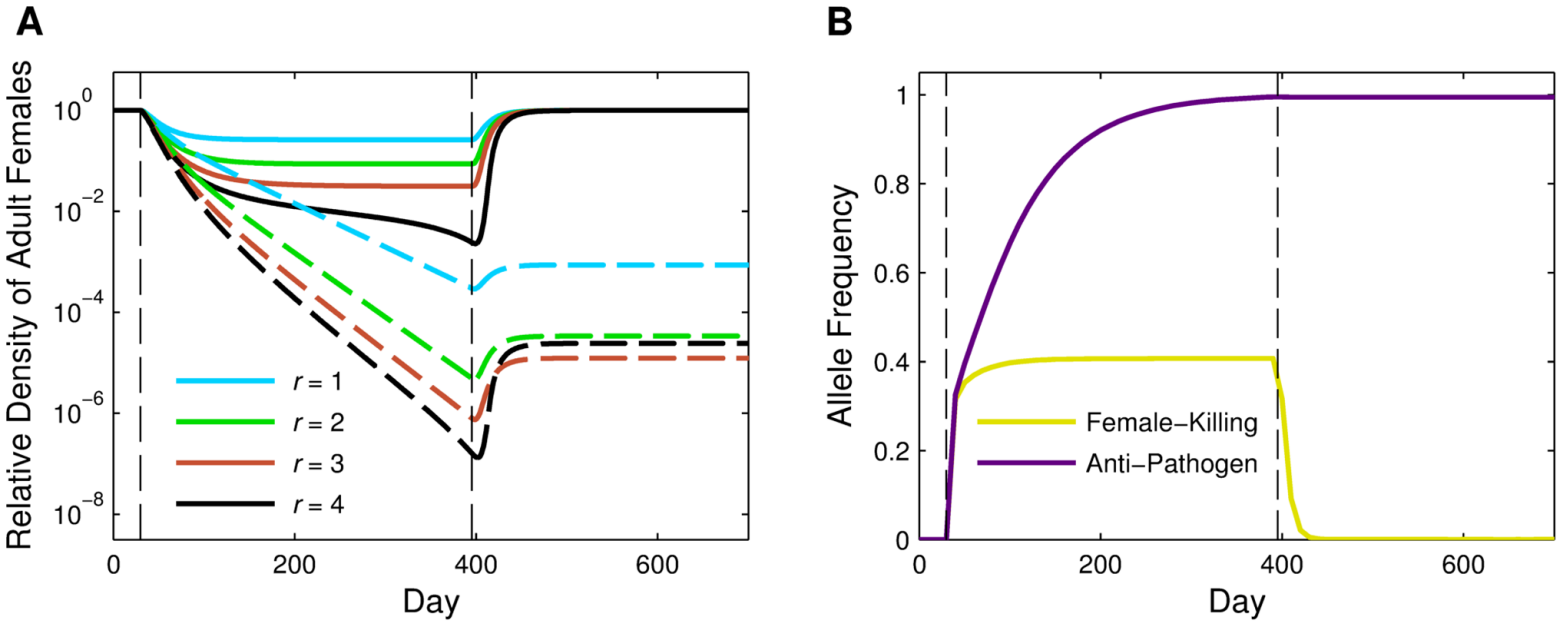
General R&R dynamics. Dynamics of an *Ae. aegypti* population when continuous male-only R&R releases occur for one year (*T* = 365). (A) Relative female population density for releases occurring at four different release ratios: *r* = 1 (blue), *r* = 2 (green), *r* = 3 (brown), and *r* = 4 (black). Dashed lines indicate the relative density of competent vectors, and solid lines represent the relative density of the total adult female population. Solid lines also indicate the relative density of the total (and thus competent) adult female population during FK releases. Density is relative to the density of the total adult female population before releases begin. Note the vertical axis is on a log scale. (B) Juvenile FK (yellow) and AP (purple) allele frequencies for a 2∶1 (*r* = 2) release. For both panels, the first vertical dashed line represents the first day of release (*t* = 30) and the second vertical dashed line represents the last day of release (*t* = 395). All other parameter values are the default values listed in Table 2.

We first considered a year-long release of R&R males. Male-only releases of GPM strains are generally preferred because released males do not contribute to additional population growth nor do they contribute to disease transmission [46]. For each of the four different release ratios (*r* = 1, *r* = 2, *r* = 3, *r* = 4), once releases of R&R males began, the density of adult females decreased, with the density of competent vectors decreasing at a more rapid rate than that of total females. This more rapid decrease of competent vectors was due to the increase in the AP allele frequency (Figure 1). When releases ended, the female population density recovered to the pre-release size, but the competent vectors, even after increasing slightly in density once releases ended, remained at a negligibly low density (Figure 1A). The juvenile FK allele frequency increased once releases began, but the FK allele was removed from the population after releases of R&R mosquitoes ended (Figure 1B shows allele frequencies for the case when *r* = 2). The long-term frequency of the AP allele depended upon the release ratio as well as the duration of release.

As the release ratio increased, the reduction in the adult female population density during releases increased. However, the marginal long-term reduction in competent females resulting from increasing the release ratio declined as this ratio increased, with high release ratios eventually resulting in higher densities of competent vectors than lower release ratios. For example, there was a greater difference in the reduction of competent vector density (log scale) between *r* = 2 and *r* = 1 than there was between *r* = 3 and *r* = 2, and for *r* = 4, the long-term density of competent vectors was actually higher than when *r* = 3 (Figure 1A). At high release ratios, the population was inundated with R&R males, and once the population was reduced, the FK and AP genes were almost always inherited together, resulting in very high linkage disequilibrium. Even if the competent vector population density was very low at the end of releases, some of the few females that remained in the population were likely to be competent vectors because most of the females that inherited the AP gene also inherited the FK gene.

In the absence of fitness costs associated with the AP allele, an R&R release had the same impact on total population density as the corresponding FK release. In the latter, however, all adult females were competent vectors: the impact of FK on competent vectors was identical to the impact of R&R on the total number of adult females (solid lines in Figure 1A). Consequently, an R&R release had a faster and longer lasting impact on the competent vector population than the corresponding FK release (compare solid and dashed lines in Figure 1A).

### Release Ratio and Duration

**Figure 2:**
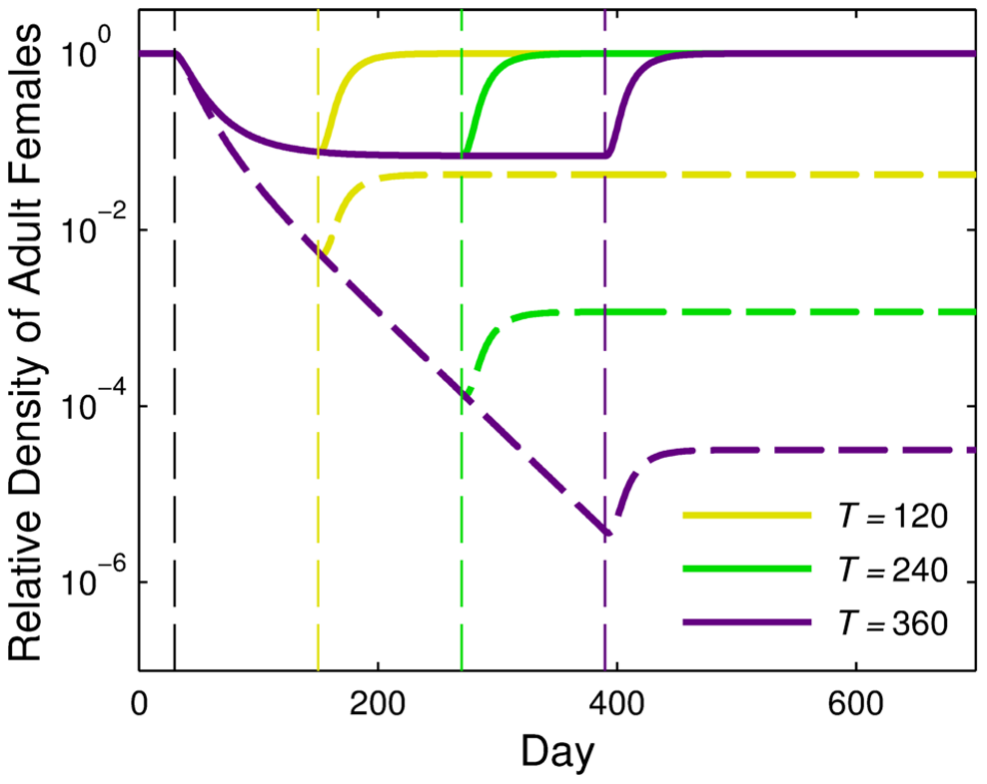
Release duration. Relative adult female population density when continuous male-only R&R releases occur at a 2∶1 (*r* = 2) release ratio for different release durations. Dashed lines indicate the relative density of the competent vectors, and solid lines indicate the relative density of the total adult female population. Each release begins on day 30, and release durations are *T* = 120 (yellow), *T* = 240 (green), and *T* = 360 (purple) days. The black vertical dashed line marks the beginning of releases, and the end of each release is indicated by a vertical dashed line of corresponding color. All other parameter values are the default values listed in Table 2. Note that the vertical axis is on a log scale.

We next considered releasing R&R mosquitoes for different periods of time. For each duration (*T* = 120, *T* = 240, *T* = 360), we simulated releases at a 2∶1 weekly release ratio. As the duration of the release increased, the density of competent vectors remaining when releases ended was lower (Figure 2); however, the reduction in total adult female population density was the same throughout the release period. This was due to the effects of density dependence. Because density dependence was strong, releases at a 2∶1 release ratio, regardless of the duration of the release, could not drive the population to extinction and instead reduced the total population to a new intermediate equilibrium density. For a weaker form of density dependence (or a higher release ratio), the total female population density may not have this intermediate equilibrium (see Text S2 and Figure S1).

**Figure 3:**
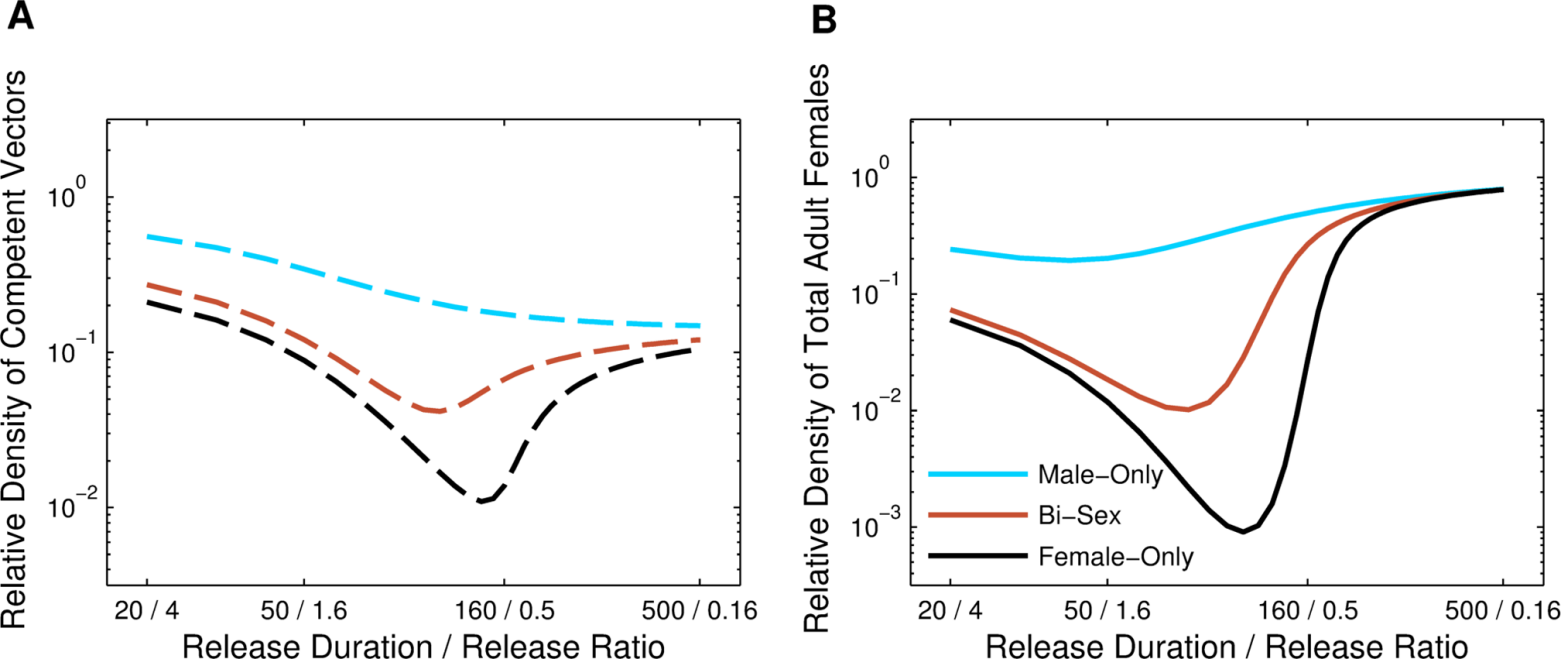
Release ratio and release duration. Relative adult female population density following releases of R&R individuals with release scenarios involving different combinations of release ratios and durations. (A) Relative density of competent vectors is measured once the total population returns to its pre-release density following male-only (blue), bi-sex (brown), and female-only (black) releases. (B) Minimum relative density of total adult females (not including released females) is measured on the day in which the minimum occurs for corresponding release scenarios. The horizontal axis for both panels is labeled as release duration/release ratio, with release durations increasing from left to right but release ratios decreasing from left to right. Each scenario results in the release of the same total number of mosquitoes. All other parameter values are the default values listed in Table 2. Note that both axes are on a log scale.

Thus far, we have shown that the magnitude of reduction of total and competent female population densities depended upon the release ratio and release duration. In each of the previous cases, a different total number of R&R individuals was released depending on the combination of release ratio and release duration. We examined the reduction in competent and total female densities for different combinations of the weekly release ratio and duration that resulted in the same total number of R&R mosquitoes being released. Releases ranged from *r* = 4 for 20 days to *r* = 0.16 for 500 days, each totaling 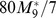 transgenic individuals. We measured the competent vector density once the total population returned to the pre-release density (Figure 3A) and the total adult female population density (Figure 3B) at the time at which the total female population density reached a minimum (note the minima in the curves in Figures 1 and 2). Scenarios in which male-only releases were conducted over longer periods resulted in greater reductions of competent vector population densities than shorter, more intense, releases (blue lines, Figure 3A). The reduction in the density of the total female population was greatest for release durations and ratios that were intermediate among the combinations we considered (blue lines, Figure 3B). The release ratio and duration at which the intermediate optimum occurred depended upon the strength of density dependence (see Text S2 and Figure S2).

### Releases Including Females

**Figure 4:**
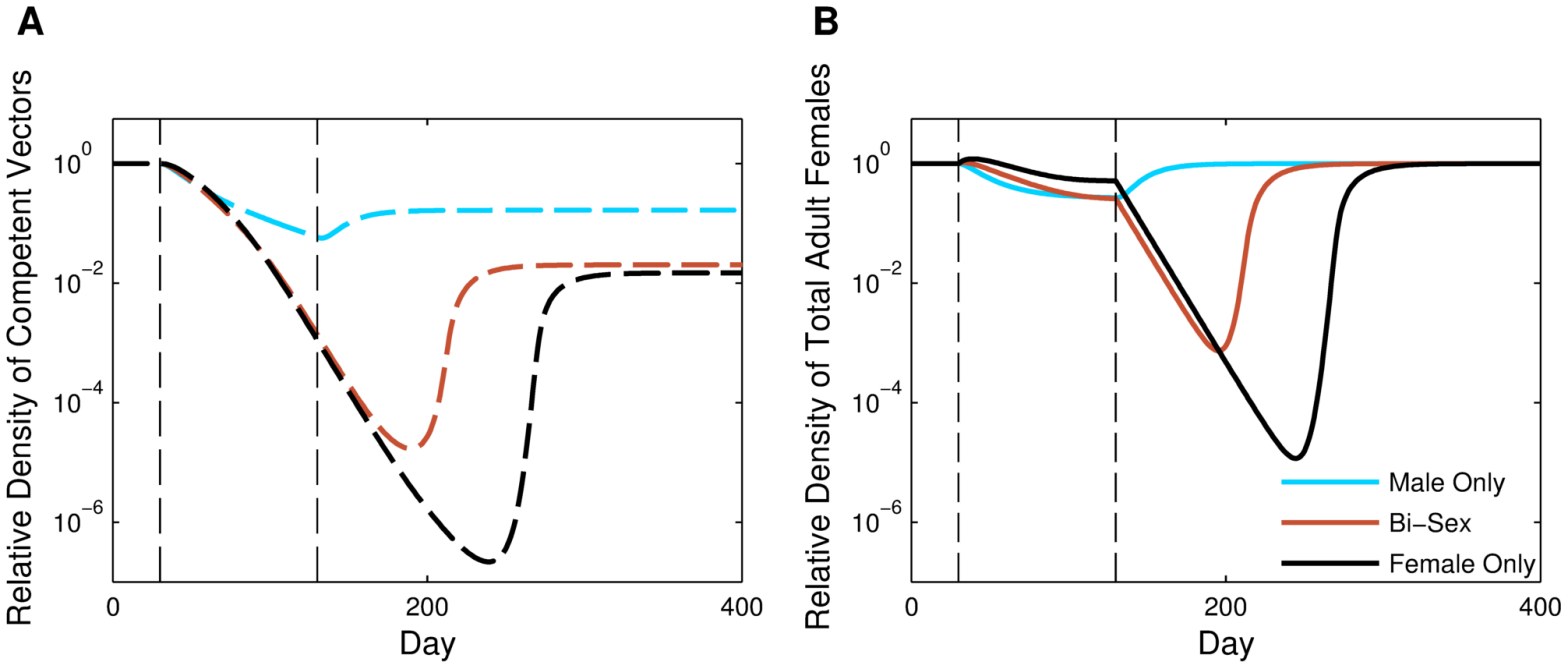
Releases including females. Relative female population density when releases are conducted at a 1∶1 (*r* = 1) release ratio for *T* = 100 days with releases of only males (blue), males and females (brown), and females only (black). (A) Relative density of competent vectors. (B) Relative density of total adult females (including released females). All other parameter values are the default values listed in Table 2. Note the vertical axis is on a log scale.

Because R&R females are assumed to be incapable of transmitting disease, we considered the option of bi-sex or female-only releases and compared the results to male-only releases. We simulated male-only, bi-sex, and female-only releases for *T* = 100 days at a release ratio of *r* = 1. If carried out for longer periods of time, bi-sex or female-only releases at this release ratio typically led towards population extinctions (see Text S2 and Figure S3). For the bi-sex case, the releases were of the same total number of individuals as in the single-sex releases, but half of the released individuals were female and half were male. In general, female-only releases resulted in the greatest reduction in the competent vector population density (Figure 4A). Female-only releases also led to the most reduction in the total adult female population density during the transient period (i.e., between the time releases began and the time when the total population was near equilibrium density again), even though when releases first began the total female population density increased noticeably due to the introduction of additional females. When we compared male-only, female-only, and bi-sex releases for release scenarios that arise from different combinations of release ratios and durations with the same total number of released mosquitoes (Figure 3), we found that for all combinations considered, female-only releases were the most effective at reducing the density of competent and total adult females (note that the density of total adult females does not include released females); however, as R&R individuals were released for longer periods of time (at lower release rates), the differences in the impacts of the three different types of releases became less noticeable (Figure 3). The combination of release duration and release ratio that resulted in the maximum reduction in total adult female density differed for each release type (Figure 3B). As with male-only releases, the intermediate optimum for each release type resulted from the trade-off between the release ratio and density-dependence effects. Bi-sex and female-only releases, however, had intermediate optimums for longer, less intense releases because releases including females had a stronger impact at lower release ratios than male-only releases. In contrast to male-only releases, intermediate combinations of release ratio and duration for bi-sex and female-only releases led to the greatest reduction in competent vectors as well (Figure 3A).

### Effects of Fitness Costs

**Figure 5:**
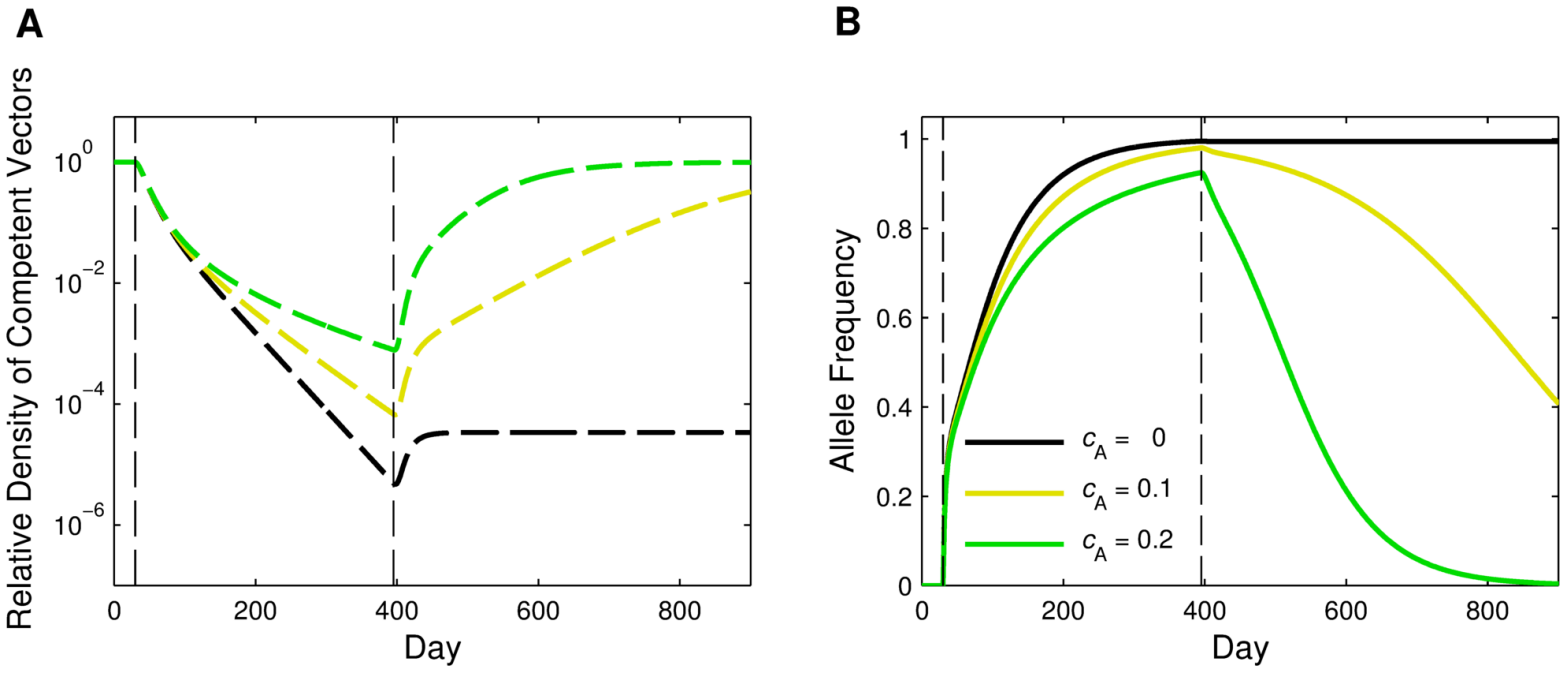
Fitness cost. Dynamics of an *Ae. aegypti* population subject to continuous male-only R&R releases at a 2∶1 (*r* = 2) release ratio for one year (*T* = 365) when there is an additive fitness cost associated with the AP allele. The fitness costs considered here are *c*_A_ = 0 (black), *c*_A_ = 0.1 (yellow), and *c*_A_ = 0.2 (green), where *c*_A_ is the fitness cost of carrying two AP alleles. (A) Relative density of competent vectors. Note the vertical axis is on a log scale. (B) AP allele frequency in the juvenile population. All other parameter values are the default values listed in Table 2.

If fitness costs are associated with carrying the AP gene, the transgene is not expected to remain in the population indefinitely after R&R releases end. We examined the impacts of fitness costs associated with the AP gene by simulating year long male-only R&R releases at a 2∶1 release ratio when *c*_A_ = 0.1 and *c*_A_ = 0.2. For higher *c*_A_, the AP gene was not able to reach as high of a frequency and went extinct more quickly. Increased fitness cost of the AP gene also led to less reduction in the competent vector population density, both during releases and after releases ended (Figure 5). However, even with *c*_A_ = 0.2, there was more rapid reduction in competent vectors than seen with releases of FK only males (compare to the solid green line in Figure 1A).

## Discussion

Using a relatively simple deterministic model, we theoretically assessed the utility of an R&R strategy, basing our analysis on hypothetical releases of the transgenic strain into a population of the dengue vector *Ae. aegypti*. We showed that repeated releases of R&R mosquitoes led to reductions in total population density, but more importantly, to both transient and long-term reductions in the density of competent vectors. In fact, within the system modeled, if the release ratio and release duration are chosen carefully, competent female vectors could effectively be eliminated from the population entirely.

We compared the R&R strategy to the FK strategy, in which transgenic mosquitoes carrying only the FK component of the R&R strain were released. We showed that in the absence of fitness costs associated with the AP gene the reduction in the total population was the same for the FK and R&R strategies throughout releases for equivalent release ratios, but the reduction in total competent vectors was much greater with the R&R strategy during releases as well as following the end of releases. Even when there was a modest fitness cost associated with the AP gene, the transient reduction in competent vectors was greater with R&R than with FK alone. This suggests that for populations in which elimination is not feasible by either strategy, R&R is superior in offering insurance against a resurgent pathogen if a program is less effective at population reduction than planned, or if the cost of sustaining releases indefinitely is not acceptable.

We showed that bi-sex and female-only releases were more effective at reducing densities of females than comparable male-only releases. In bi-sex releases, the first generation of offspring could receive two copies of both the FK and AP alleles. This facilitated both a more rapid increase in the AP allele frequency and a greater decrease in the population than comparable levels of male-only releases. When R&R females were released, whether in bi-sex or female-only releases, they quickly dominated the population of mating females as the FK allele spread in the population so that most offspring were those of the R&R females and had both FK and AP alleles. Moreover, releases of females resulted in increased offspring production, which in turn led to additional larval competition and thus higher rates of larval mortality. The combination of the more rapid rise in the frequency of the FK and AP genes and the increased larval mortality resulted in greater population declines that affected both competent and total female population density than comparable male-only releases.

We showed that while increased ratios and increased durations of R&R male-only releases led to greater reduction in competent vectors, release duration was the more important factor when density dependence was strong. Similar to results seen in previous modeling exercises for other GPM strategies, completely inundating a population with R&R males for a short period of time was not optimal primarily because the number of wild-type females was limited [33], [47]. Additionally, releasing at high release ratios could have resulted in linkage disequilibrium when population density was low, causing the FK and AP alleles to be inherited together too frequently. This led to increased mortality of vectors that would otherwise be incompetent, and thus many of the vectors remaining when the total population density was low were competent vectors. We found that the greatest reduction in the total female population density for male-only, bi-sex, and female-only releases, as well as the greatest reduction in competent vector density for bi-sex and female-only releases, was observed for intermediate combinations of release ratio and duration. This is due to the interaction between density-dependent population regulation and the population reduction caused by the FK gene. High-intensity releases conducted over a short period of time became wasteful because the marginal benefit from releasing more R&R individuals waned as the frequency of the FK allele increased in the population. On the other hand, when low-intensity releases were conducted over a long period of time, each release had less impact because density dependence had time to counteract any population reduction. The long-term density of competent vectors for R&R releases reflected the impact of both FK and AP components of the strain. For the release of a neutral AP only strain (i.e., without the FK component), the ultimate density of competent vectors would decrease monotonically with increasing release duration (A.L. Lloyd, unpublished results).

For the strong density dependence considered in the main text of this study, we observed a non-monotonic pattern in the long-term density of competent vectors following both female-only and bi-sex releases, but a strictly decreasing density with increased release duration for male-only releases. For weaker density dependence, the ultimate densities of competent vectors after male-only releases also exhibited this non-monotonic pattern (refer to Text S2 and Figure S2).We also found that under conditions of weaker density dependence the R&R releases still caused a more rapid reduction in the density of competent vectors than was seen with comparable FK releases (compare the two panels of Figure S1). Furthermore, because a population regulated by weak density dependence could not recover from population reduction as quickly as one which is regulated by stronger density dependence, there was a greater reduction in total female population density for populations regulated by weak density dependence as a result of R&R releases (see the right panel of Figure S1). These results, although obtained from a simple treatment of density dependence, underscore the importance of understanding the density-dependent processes underlying population dynamics before beginning any vector control program.

Despite our findings that releases including females are expected to result in greater reduction in competent vectors, the introduction of females may face opposition from local communities because wild females bite and transmit disease. Although all released females and their offspring should be incapable of transmitting disease, the potential for increased biting nuisance or even a small chance of disease transmission could make female releases less acceptable than male releases. However, the recent release of female *Ae. aegypti* in Australia infected with *Wolbachia* [48] makes the case that female releases could be acceptable when the released individuals are not competent vectors.

All GPM strategies can be hampered if there is a fitness disadvantage associated with the inserted transgenes. Such fitness costs can manifest themselves in a number of ways, including reductions in attractiveness to mates, fecundity, and survival. Here, we considered an additive fitness cost associated with the AP gene that reduced the survival of offspring during the egg stage. We showed that higher fitness costs slowed the rate of competent vector population reduction and reduced the amount of time that the AP allele remained in the population once releases ended. However, even with a substantial fitness cost to the AP gene, the R&R releases reduced the density of competent vectors more rapidly than the FK releases without a fitness cost. If there are any fitness disadvantages associated with the AP gene of the R&R strain, releases would need to be conducted continuously, perhaps in the form of smaller maintenance releases. Our results highlight that in order for an R&R strategy to reach its full promise as a solution to the logistical difficulties of an FK strategy, minimizing any such fitness costs is important.

As with other strategies, the efficacy of R&R could be reduced by immigration of wild-type mosquitoes. The impact of immigration on population-wide vector competence depended on the magnitude of the rate of immigration (see Text S2). Higher wild-type immigration rates accelerated the recovery of competent vector densities to pre-release equilibrium levels shortly after R&R releases ended. If immigration rates were lower, however, much more time was needed for the competent vector population to increase to the pre-release density (Figure S4). The predicted impacts of wild-type immigration on the success of an R&R strategy are similar to those expected based on previous modeling studies of population reduction strategies [31], [49]; however, a more detailed exploration of the potential impacts of immigration on an R&R strategy is warranted before R&R releases are conducted.

In this paper, we demonstrated that an R&R strategy is likely to be preferable to an FK strategy under a variety of scenarios due to its ability to cause a greater, sustainable reduction in competent vectors. We emphasize this comparison because FK strategies and related GPM population reduction strategies have seen notable progress, and large-scale releases are becoming increasingly possible [26]–[27], [50]. Although strategies for introducing AP genes without the aid of gene drive have been considered [51], they have received less attention; however, developments in molecular technology that lead to sustained inhibition of pathogen transmission via transgenes and minimal fitness costs associated with transgenes could make such AP-only strategies more feasible. While it is beyond the scope of this paper, R&R releases could potentially be evaluated against AP-only strategies as well strategies that combine FK, AP, and R&R releases.

The R&R strategy led to greater long term reduction in competent vectors because of the combination of population reduction and replacement genes. Most previous studies have focused either on population reduction or population replacement. Some population replacement strategies would have some degree of transient population reduction due to the genetic load imposed during the replacement process (e.g., *Wolbachia* [52], *Semele* [16]), and it has been proposed that insecticides be used to reduce populations before beginning a population replacement strategy. In contrast, the R&R strategy is specifically designed to cause population reduction while simultaneously spreading an AP gene to reduce competent vector density, and continuous releases of R&R mosquitoes would lead to continuous reduction. Although many gene drive strategies have been proposed and theoretically assessed, successful implementation of these strategies for disease vectors has proven difficult. An R&R strategy, however, could take advantage of the advances that have been made in the independent development of AP and FK strategies.

Although the study we presented here has demonstrated the potential utility of an R&R strategy, we emphasize that the model used is very general and is only intended to provide an introduction to, and broad overview of, a novel strategy that can be useful in the fight against vector-borne diseases. As with any vector control strategy, a more thorough species-specific assessment should be considered before implementation of an R&R strategy. The role of stochastic effects, spatial heterogeneity, immigration, and abiotic factors such as meteorological fluctuations in the dynamics of a population subject to R&R releases should be carefully evaluated using models that incorporate more details than have been considered here.

## Supporting Information

Figure S1 **R&R and density dependence.** Dynamics of an *Ae. aegypti* population subject to continuous male-only R&R releases at a 1∶1 (*r* = 1) release ratio for 120 days for different strengths of density dependence. (A) Relative density of competent vectors. (B) Relative density of total adult females. Note that this panel also indicates the relative density of the total (and thus competent) adult female population during FK releases. For both panels, the first vertical dashed line represents the first day of release (30) and the second vertical dashed line represents the last day of release (150). All other parameter values are the default values listed in Table 2 of the main text. Note the vertical axis for both panels is on a log scale.
(TIF)

Figure S2 **Density dependence and release ratio and duration.** Relative adult female population density following releases of R&R males into populations regulated by different strengths of density dependence with release scenarios involving different combinations of release ratios and durations. (A) Relative density of competent vectors is measured once the total population returns to its pre-release density following releases. (B) Minimum relative density of total adult females is measured on the day in which the minimum occurs for corresponding release scenarios. The horizontal axis for both panels is labeled as release duration/release ratio, with release durations increasing from left to right but release ratios decreasing from left to right. Each scenario results in the release of the same total number of male mosquitoes. All other parameter values are the default values listed in Table 2. Note that both axes are on a log scale.
(TIF)

Figure S3 **Duration of female-only releases.** Relative total adult female population density when continuous male-only R&R releases occur at a 1∶1 (*r* = 1) release ratio for different release durations. Each release begins on day 30, and release durations are *T* = 100 (green), *T* = 110 (yellow), and *T* = 120 (black) days. The black vertical dashed line marks the beginning of releases, and the end of each release is indicated by a vertical dashed line of corresponding color. All other parameter values are the default values listed in Table 2. Note that the vertical axis is on a log scale.
(TIF)

Figure S4 **Immigration of wild-type juveniles.** Relative total adult female population density (solid lines) and relative competent vector density (dashed lines) when continuous male-only R&R releases occur at a 2∶1 (*r* = 2) release ratio for *T* = 100 days in the presence of wild-type juvenile immigration. The black vertical dashed lines mark the beginning and end of releases. Immigration rates are defined in terms of a fraction of the equilibrium juvenile density per day: No immigration (blue), 0.001 J_9_^*^ (green), 0.01 J_9_^*^ (brown), and 0.05 J_9_^*^ (purple). All other parameter values are as in Table 2. Note that the vertical axis is on a log scale.
(TIF)

Text S1 **Equilibrium Analysis.** A brief mathematical analysis of the wild-type equilibrium for this model.
(PDF)

Text S2 **Further Exploration.** Discussion on the effects of density dependence and immigration on R&R releases and a note on the effects of long release duration of releases including females.
(PDF)
