# Supplementary figures and images for: A Reduce and Replace Strategy for Suppressing Vector-Borne Diseases: Insights from a Deterministic Model
Source: PLoS One. 2013 Sep 4;8(9):e73233. doi: 10.1371/journal.pone.0073233 (PMC3762895; doi:10.1371/journal.pone.0073233)

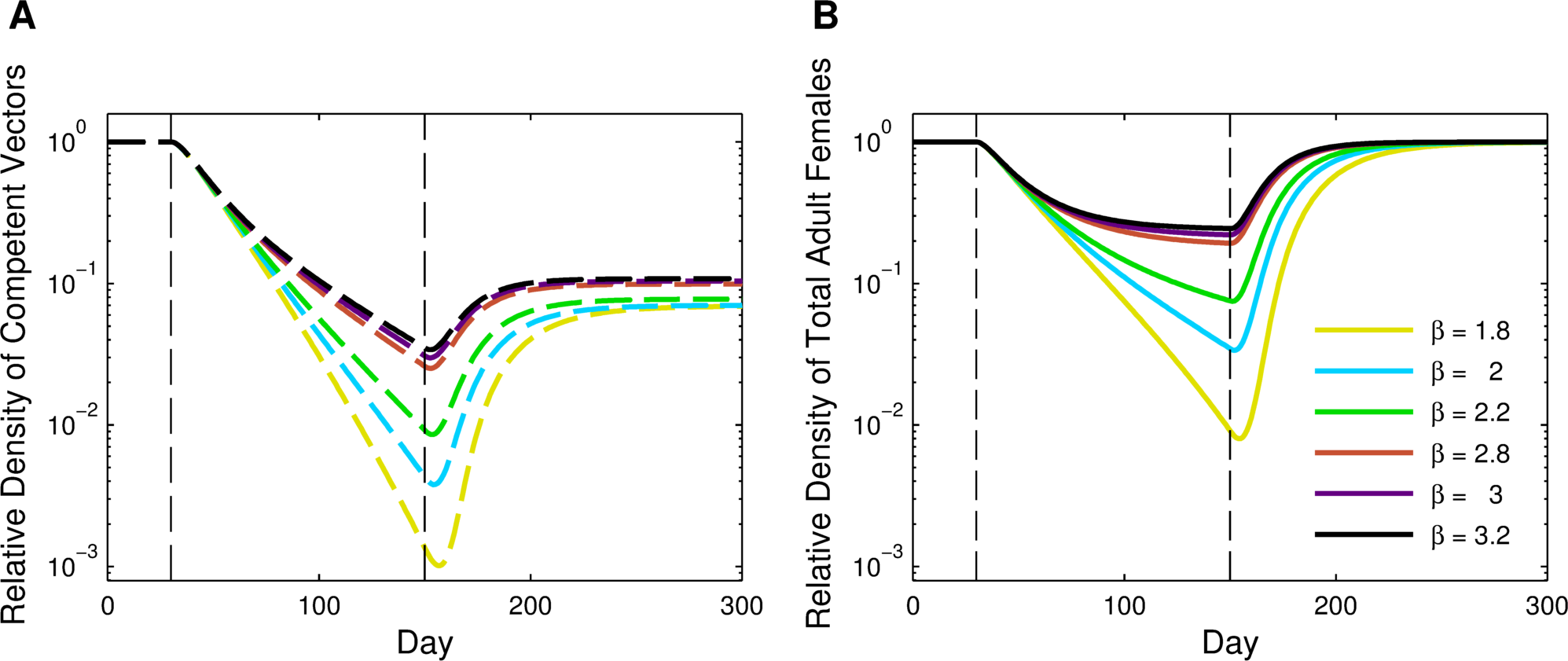

Supplement: Figure S1 — R&R and density dependence. Dynamics of an Ae. aegypti population subject to continuous male-only R&R releases at a 1∶1 (r = 1) release ratio for 120 days for different strengths of density dependence. (A) Relative density of competent vectors. (B) Relative density of total adult females. Note that this panel also indicates the relative density of the total (and thus competent) adult female population during FK releases. For both panels, the first vertical dashed line represents the first day of release (30) and the second vertical dashed line represents the last day of release (150). All other parameter values are the default values listed in Table 2 of the main text. Note the vertical axis for both panels is on a log scale. (TIF) [file pone.0073233.s001.tif]

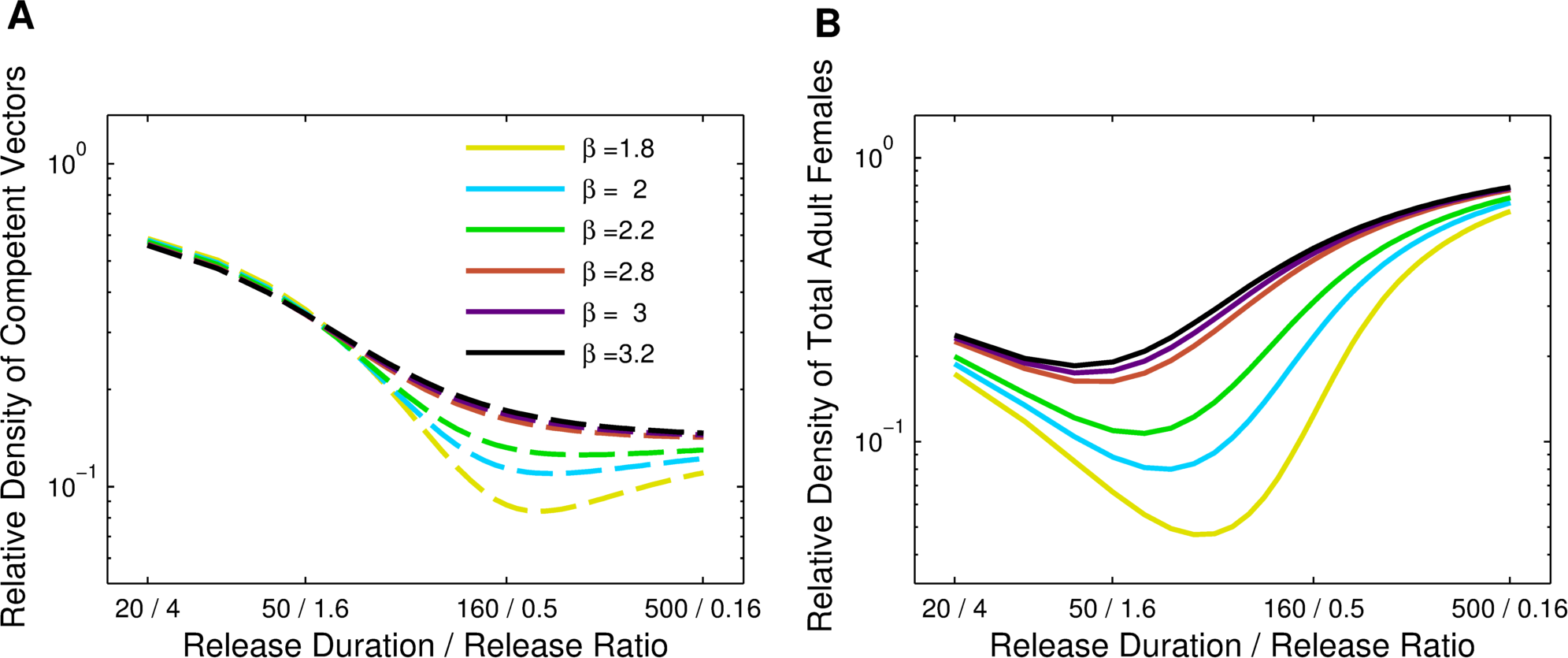

Supplement: Figure S2 — Density dependence and release ratio and duration. Relative adult female population density following releases of R&R males into populations regulated by different strengths of density dependence with release scenarios involving different combinations of release ratios and durations. (A) Relative density of competent vectors is measured once the total population returns to its pre-release density following releases. (B) Minimum relative density of total adult females is measured on the day in which the minimum occurs for corresponding release scenarios. The horizontal axis for both panels is labeled as release duration/release ratio, with release durations increasing from left to right but release ratios decreasing from left to right. Each scenario results in the release of the same total number of male mosquitoes. All other parameter values are the default values listed in Table 2. Note that both axes are on a log scale. (TIF) [file pone.0073233.s002.tif]

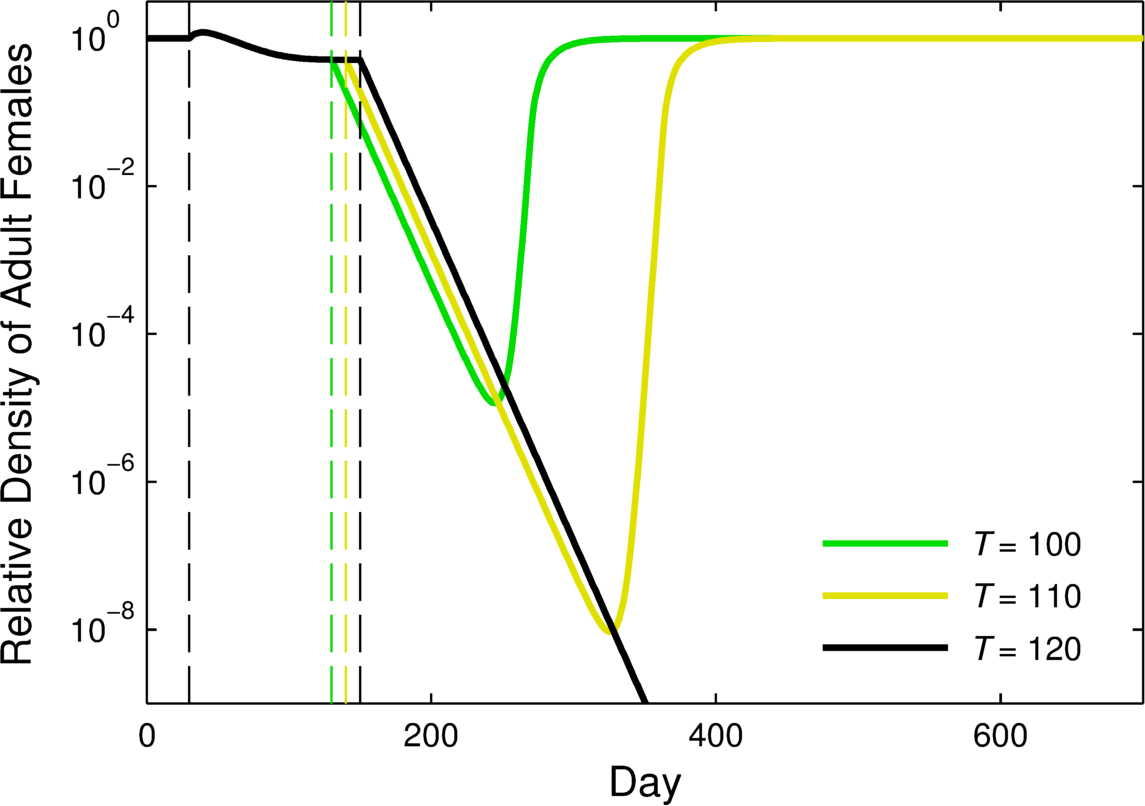

Supplement: Figure S3 — Duration of female-only releases. Relative total adult female population density when continuous male-only R&R releases occur at a 1∶1 (r = 1) release ratio for different release durations. Each release begins on day 30, and release durations are T = 100 (green), T = 110 (yellow), and T = 120 (black) days. The black vertical dashed line marks the beginning of releases, and the end of each release is indicated by a vertical dashed line of corresponding color. All other parameter values are the default values listed in Table 2. Note that the vertical axis is on a log scale. (TIF) [file pone.0073233.s003.tif]

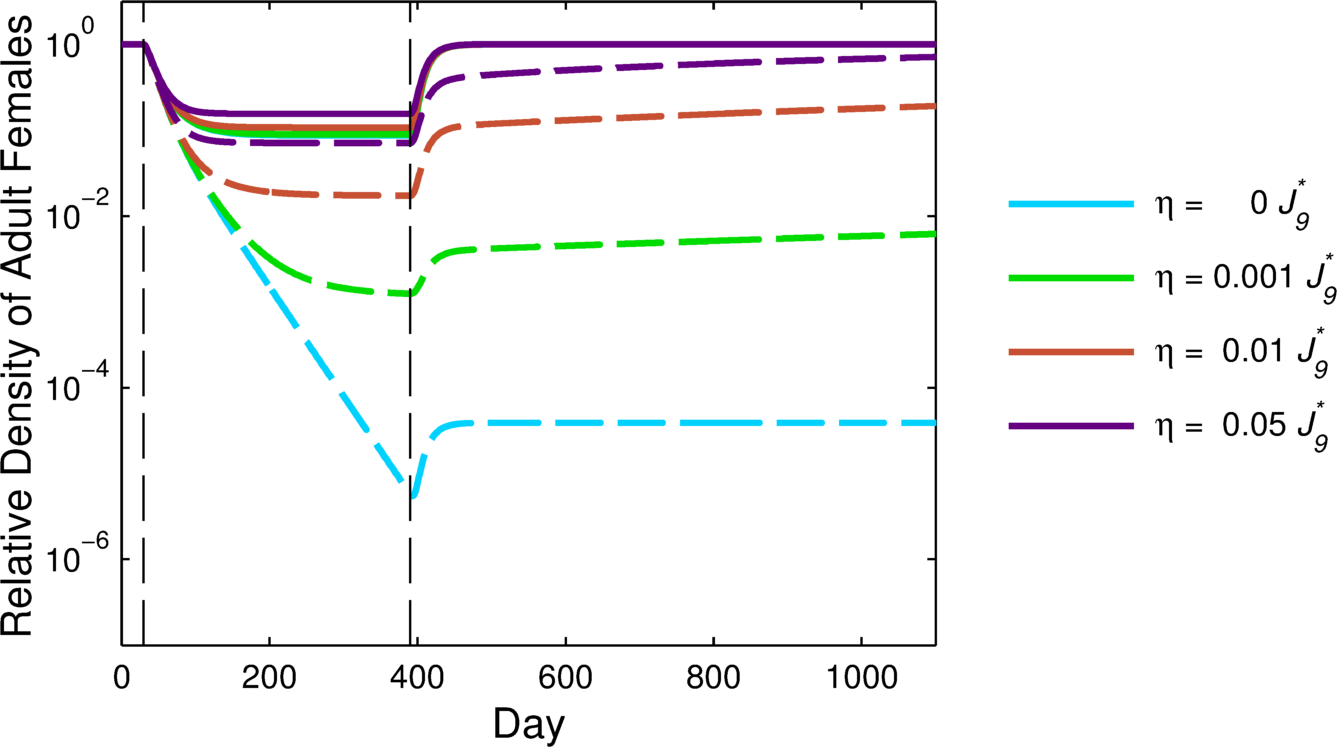

Supplement: Figure S4 — Immigration of wild-type juveniles. Relative total adult female population density (solid lines) and relative competent vector density (dashed lines) when continuous male-only R&R releases occur at a 2∶1 (r = 2) release ratio for T = 100 days in the presence of wild-type juvenile immigration. The black vertical dashed lines mark the beginning and end of releases. Immigration rates are defined in terms of a fraction of the equilibrium juvenile density per day: No immigration (blue), 0.001 J9* (green), 0.01 J9* (brown), and 0.05 J9* (purple). All other parameter values are as in Table 2. Note that the vertical axis is on a log scale. (TIF) [file pone.0073233.s004.tif]
