# Supplementary material for: A Reduce and Replace Strategy for Suppressing Vector-Borne Diseases: Insights from a Deterministic Model
Source: PLoS One. 2013 Sep 4;8(9):e73233. doi: 10.1371/journal.pone.0073233 (PMC3762895; doi:10.1371/journal.pone.0073233)
Supplement: Text S1 — Equilibrium Analysis. A brief mathematical analysis of the wild-type equilibrium for this model. (PDF) [file pone.0073233.s005.pdf]

A Reduce and Replace strategy for suppressing vector-borne diseases: insights from a deterministic model

Michael A. Robert, Kenichi Okamoto, Alun L. Lloyd, Fred Gould

Supporting Text S1. Equilibrium Analysis.

In the main text, we describe the development of a system of ordinary differential equations that we use to simulate population dynamics and population genetics of *Aedes aegypti* following the introduction of an R&R strain into a wild-type population. Analysis of this model is limited by the model complexity; however, we are able to obtain equilibrium population density of the wild-type population in the absence of transgenic releases.

In a completely wild-type population, the system

$$\dot{J}_i = B_i(t) - \mu_J J_i - J_i \left(\alpha \sum_g J_g \right)^{\beta-1} - \nu J_i$$

$$\dot{F}_i = \frac{1}{2}\nu \gamma_i J_i - \mu_F F_i + u_i^F$$

$$\dot{M}_i = \frac{1}{2}\nu J_i - \mu_M M_i + u_i^M \tag{1}$$

$$B_i(t) = w_i \lambda \sum_m F_m(t) \sum_n Pr(i|m,n) \frac{M_n(t)}{\sum_g M_g(t)}$$

for $i = 1...9$, where $i = 9$ represents the wild-type genotype, reduces to

$$\dot{J}_9 = \lambda F_9 - \mu_J J_9 - \alpha^{\beta-1} J_9^{\beta} - \nu J_9$$

$$\dot{F}_9 = \frac{1}{2}\nu J_9 - \mu_F F_9 \tag{2}$$

$$\dot{M}_9 = \frac{1}{2}\nu J_9 - \mu_M M_9.$$

Here, $\dot{M}_9$ is decoupled from the system, so we can analyze the reduced system

$$\dot{J}_9 = \lambda F_9 - \mu_J J_9 - \alpha^{\beta-1} J_9^{\beta} - \nu J_9$$

$$\dot{F}_9 = \frac{1}{2}\nu J_9 - \mu_F F_9 \ . \tag{3}$$

This system has a trivial equilibrium at $(J_9^{(1)}, F_9^{(1)}) = (0,0)$, and one non-trivial equilibrium at

$$J_9^{(2)} = \frac{1}{\alpha}\left(\frac{\nu\lambda}{2\mu_F} - \mu_J - \nu\right)^{\frac{1}{\beta-1}}$$

$$F_9^{(2)} = \frac{\nu}{2\mu_F} J_9^{(2)} \ . \tag{4}$$

We rearrange the expression for $J_9^{(2)}$ by noting that

$$\frac{\nu\lambda}{2\mu_F} - \mu_J - \nu = (\mu_J + \nu)\left(\frac{\nu\lambda}{2\mu_F(\mu_J+\nu)} - 1\right)$$

$$= (\mu_J + \nu)(R_0 - 1) \ , \tag{5}$$

where

$$R_0 = \frac{1}{\mu_F} \cdot \frac{\lambda}{2} \cdot \frac{\nu}{\mu_J + \nu} = \frac{\nu\lambda}{2\mu_F(\mu_J+\nu)} \ . \tag{6}$$

Here, $\frac{1}{\mu_f}$ is the average lifespan of adult females, $\frac{\lambda}{2}$ is the rate of production of female offspring, and $\frac{\nu}{\mu_J+\nu}$ is the fraction of juveniles that survive to emerge as adults. Thus, R_0 is the basic reproductive

number of the population. We rewrite (4) in terms of R_0.

$$\begin{aligned} J_9^{(2)} &= \frac{1}{\alpha}\left((\mu_J+\nu)(R_0-1)\right)^{\frac{1}{\beta-1}} \\ F_9^{(2)} &= \frac{\nu}{2\mu_F} J_9^{(2)} \end{aligned} \tag{7}$$

In order for population to have a positive equilibrium (i.e., $J_9^{(2)} > 0$), $R_0 > 1$. Thus, we analyze the stability of the equilibrium only for the case when $R_0 > 1$.

To verify the stability of the equilibrium in (7), we first find the Jacobian of system (3).

$$\text{Jacobian}(J_9, F_9) = \begin{pmatrix} -\left(\mu_J + \nu + \beta(\alpha J_9)^{\beta-1}\right) & \lambda \\ \frac{1}{2}\nu & -\mu_F \end{pmatrix} \tag{8}$$

We then evaluate the Jacobian at the equilibrium in (7).

$$\mathcal{J} = \text{Jacobian}(J_9^{(2)}, F_9^{(2)}) = \begin{pmatrix} -\left(\mu_J + \nu + \beta(\mu_J+\nu)(R_0-1)\right) & \lambda \\ \frac{1}{2}\nu & -\mu_F \end{pmatrix} \tag{9}$$

We now study the eigenvalues of $\mathcal{J}$ by studying the determinant and trace of $\mathcal{J}$. The equilibrium point $(J_9^{(2)}, F_9^{(2)})$ is stable when $\text{Tr}(\mathcal{J}) < 0$ and $\det(\mathcal{J}) > 0$ (i.e., both eigenvalues of $\mathcal{J}$ must be negative). First, we calculate $\text{Tr}(\mathcal{J})$.

$$\text{Tr}(\mathcal{J}) = -(\mu_J + \nu + \beta(\mu_J+\nu)(R_0-1) + \mu_F) \tag{10}$$

Since $R_0 > 1$, and because we require μ_J, ν, β, and μ_F to be positive, $\text{Tr}(\mathcal{J}) < 0$. Next, we calculate $\det(\mathcal{J})$.

$$\det(\mathcal{J}) = \mu_F\left(\mu_J + \nu + \beta(\mu_J+\nu)(R_0-1)\right) - \frac{\lambda\nu}{2} \tag{11}$$

Rearranging the terms, we get

$$\det(\mathcal{J}) = 1 + \beta(R_0 - 1) - \frac{\lambda\nu}{2\mu_F(\mu_J + \nu)}$$

$$= 1 + \beta(R_0 - 1) - R_0 \ . \tag{12}$$

In order for $\det(\mathcal{J}) > 0$,

$$1 + \beta(R_0 - 1) - R_0 > 0$$

$$\beta(R_0 - 1) > R_0 - 1 \tag{13}$$

$$\beta > 1 \ .$$

So we have that the equilibrium (J_9^*, F_9^*) is stable when $\beta > 1$.

Equilibrium Values for Model Runs

Here, we list the values of the equilibrium density of juveniles, adult males, and adult females that are used for model runs in the main text. Note that the release size of R&R individuals is always defined as a function of the equilibrium wild-type male population density so that release rates are always relative to the population density. This allows for a general study of R&R releases in an *Ae. aegypti* population. While changes in α will result in changes in the density of the population, the qualitative results for relative density are the same.

$$J_9^* = 10118.98$$

$$F_9^* = 7083.28 \tag{14}$$

$$M_9^* = 2529.74$$
