# Supplementary material for: A Reduce and Replace Strategy for Suppressing Vector-Borne Diseases: Insights from a Deterministic Model
Source: PLoS One. 2013 Sep 4;8(9):e73233. doi: 10.1371/journal.pone.0073233 (PMC3762895; doi:10.1371/journal.pone.0073233)
Supplement: Text S2 — Further Exploration. Discussion on the effects of density dependence and immigration on R&R releases and a note on the effects of long release duration of releases including females. (PDF) [file pone.0073233.s006.pdf]

A Reduce and Replace strategy for suppressing vector-borne diseases: insights from a deterministic model

Michael A. Robert, Kenichi Okamoto, Alun L. Lloyd, Fred Gould

Supporting Text S2. Further Exploration

Density dependence

Throughout the main text, we consider a population in which the strength of density-dependent larval regulation is strong. Here, we vary the strength of density dependence by considering different values of β ($\beta = 1.8$, $\beta = 2$, $\beta = 2.2$, $\beta = 2.8$, $\beta = 3$, and $\beta = 3.2$). These results are presented in Figure S1. For lower values of β, the transient reduction in both the total female and competent vector population densities is greater than for higher values of β. This occurs because populations rebound more quickly from perturbations away from equilibrium density for higher values of β (stronger density dependence). For these higher values of β, the total adult female population reaches an intermediate equilibrium density while releases occur before returning to the pre-release density. For lower values of β, however, including $\beta = 2$ (which would correspond to the familiar logistic term), the total population density decreases towards extinction, but eventually returns to pre-release densities after releases end. If population elimination is desired in a population in which density-dependent regulation is strong, release sizes would need to be increased to ratios high enough to overcome this strong density-dependent population regulation. In these populations, R&R releases may be desired because even though population elimination is difficult to achieve, R&R releases lead to a lower density of competent vectors than FK only releases (compare each panel of Figure S1).

In the main text, we consider the reduction in competent and total female vector densities that results from different combinations of release ratio and duration. In each release, the same total number of R&R males is released. Here, we consider the effects of density dependence on release scenarios that arise from these different combinations (Figure S2). The intermediate combinations in which total population reduction is the greatest depends upon the strength of density dependence.

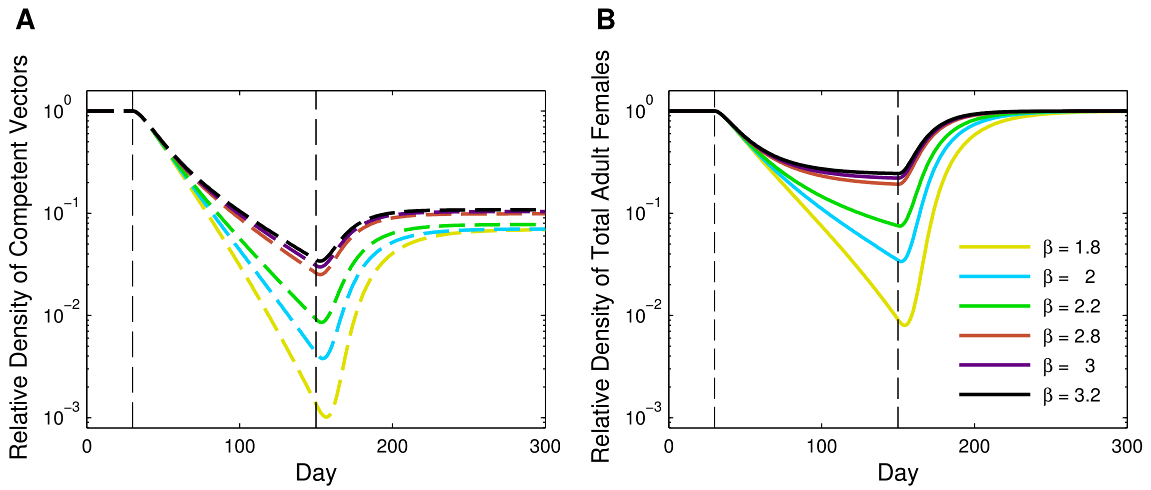

Figure S1. R&R and density dependence. Dynamics of an *Ae. aegypti* population subject to continuous male-only R&R releases at a 1:1 ($r = 1$) release ratio for 120 days for different strengths of density dependence. (A) Relative density of competent vectors. (B) Relative density of total adult females. Note that this panel also indicates the relative density of the total (and thus competent) adult female population during FK releases. For both panels, the first vertical dashed line represents the first day of release (30) and the second vertical dashed line represents the last day of release (150). All other parameter values are the default values listed in Table 2 of the main text. Note the vertical axis for both panels is on a log scale.

As density dependence gets stronger (higher values of β), population reduction is greatest at lower intermediate release durations. This is because the population is capable of rebounding much more quickly than populations with weaker density dependence. For stronger density dependence, short but very intense releases are required to maximize population reduction, and smaller release sizes over long periods of time cannot overcome the strength of density dependence.

One should also note that the shape of the curves describing the reduction in competent vectors change slightly as density-dependent population regulation strengthens. As β increases, the relative density of competent vectors changes from a non-monotonic to a monotonically decreasing curve for the combinations of release ratio and release duration considered here. For each of the non-monotonic curves, the minimum occurs for scenarios in which releases occur at low intensity over longer periods of time. As density dependence strengthens, the marginal benefit of increasing release duration is lost because the population can rebound more easily, and the smaller releases have less impact on the population.

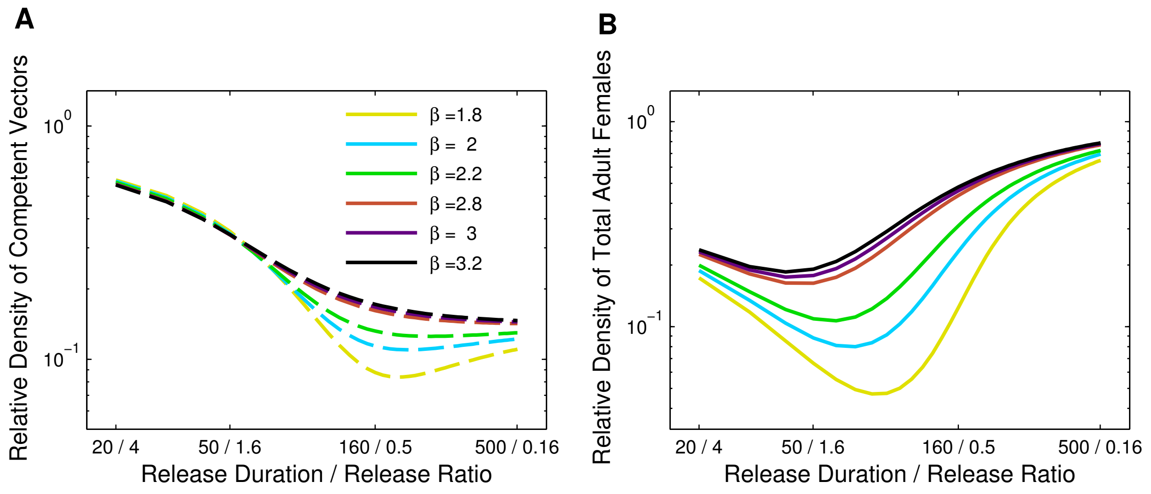

Figure S2. Density dependence and release ratio and duration. Relative adult female population density following releases of R&R males into populations regulated by different strengths of density dependence with release scenarios involving different combinations of release ratios and durations. (A) Relative density of competent vectors is measured once the total population returns to its pre-release density following releases. (B) Minimum relative density of total adult females is measured on the day in which the minimum occurs for corresponding release scenarios. The horizontal axis for both panels is labeled as release duration / release ratio, with release durations increasing from left to right but release ratios decreasing from left to right. Each scenario results in the release of the same total number of male mosquitoes. All other parameter values are the default values listed in Table 2. Note that both axes in each panel are on a log scale.

Release duration and female-only releases

In the main text, we present results from male-only, bi-sex, and female-only releases conducted at a 1:1 ratio for only 100 days. We mention there that this combination of release duration and release ratio was chosen because longer durations of releases including females typically led to population extinctions. Here, we show that the total female population density decreases rapidly as the release duration is increased when female-only releases are conducted at a 1:1 ratio (Figure S3). In fact, for the parameter set considered here, a release duration $T = 120$ days led to extinction of the population whereas a release for $T = 110$ days did not.

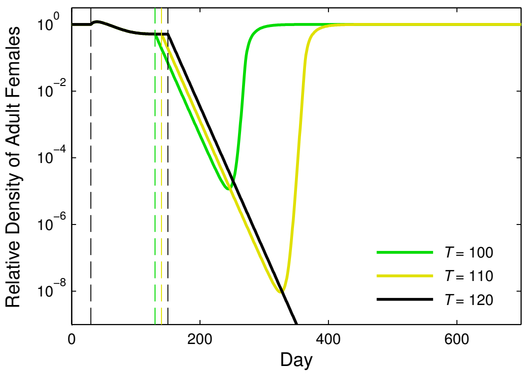

Figure S3. Duration of female-only releases. Relative total adult female population density when continuous male-only R&R releases occur at a 1:1 ($r = 1$) release ratio for different release durations. Each release begins on day 30, and release durations are $T = 100$ (green), $T = 110$ (yellow), and $T = 120$ (black) days. The black vertical dashed line marks the beginning of releases, and the end of each release is indicated by a vertical dashed line of corresponding color. All other parameter values are the default values listed in Table 2. Note that the vertical axis is on a log scale.

Wild-type immigration

Here, we study the impacts of immigration of wild-type juveniles. We assume that immigrants enter the population at a constant daily rate, η, before, during, and after R&R releases occur. We note that this changes the equation describing the dynamics of wild-type juveniles to

$$\dot{J}_9 = B_9(t) - \mu_J J_9 - J_9 \left(\alpha \sum_g J_g \right)^{\beta - 1} - \nu J_9 + \eta \ .$$

We define immigration rates as fractions of the equilibrium wild-type juvenile density per day. For example, if immigration occurs at a rate of 1% of the wild-type equilibrium density per day, then $\eta = 0.01 J_9^* \text{day}^{-1}$. We find that the impact of immigration on the density of competent vectors depends upon the magnitude of the immigration rate (Figure S4). That is, larger immigration rates lead to smaller decreases in the competent vector population than smaller immigration rates. Furthermore, larger immigration rates result in the competent vector density returning towards the pre-release equilibrium much more quickly than smaller immigration rates. For the smallest immigration rate we consider here, the competent vector density does not increase much, even hundreds of days after R&R releases end, whereas the competent vector population is near pre-release equilibrium soon after R&R releases end for the largest immigration rates we consider. This

is in part due to the inability to reduce the competent vector population as much in the presence of larger immigration rates. Although immigration does hinder R&R releases from leading to the same reduction in competent vectors as is observed without immigration, R&R releases still lead to more reduction in competent vectors than FK releases, regardless of the immigration rate (compare solid and dashed lines in Figure S4).

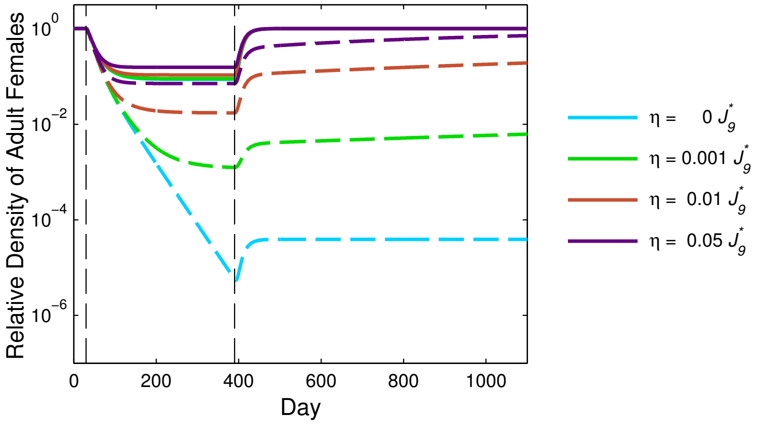

Figure S4. Immigration of wild-type juveniles. Relative total adult female population density (solid lines) and relative competent vector density (dashed lines) when continuous male-only R&R releases occur at a 2:1 ($r = 2$) release ratio for $T = 100$ days in the presence of wild-type juvenile immigration. Solid lines also indicate the relative density of the total (and thus competent) adult female population during FK releases. The black vertical dashed lines mark the beginning and end of releases. Immigration rates are defined in terms of a fraction of the equilibrium juvenile density per day: No immigration (blue), 0.001 J_9^* (green), 0.01 J_9^* (brown), and 0.05 J_9^* (purple). All other parameter values are the default values listed in Table 2. Note that the vertical axis is on a log scale.
